# Beneficial Effect and Potential Risk of *Pantoea* on Rice Production

**DOI:** 10.3390/plants11192608

**Published:** 2022-10-04

**Authors:** Luqiong Lv, Jinyan Luo, Temoor Ahmed, Haitham E. M. Zaki, Ye Tian, Muhammad Shafiq Shahid, Jianping Chen, Bin Li

**Affiliations:** 1 State Key Laboratory of Rice Biology and Ministry of Agriculture Key Laboratory of Molecular Biology of Crop Pathogens and Insects, Key Laboratory of Biology of Crop Pathogens and Insects of ZhejiangProvince, Institute of Biotechnology, Zhejiang University, Hangzhou 310058, China; 2 Department of Plant Quarantine, Shanghai Extension and Service Center of Agriculture Technology, Shanghai 201103, China; 3 Horticulture Department, Faculty of Agriculture, Minia University, El-Minia 61517, Egypt; 4 Applied Biotechnology Department, University of Technology and Applied Sciences-Sur, Sur 411, Oman; 5 Department of Plant Sciences, College of Agricultural and Marine Sciences, Sultan Qaboos University, Al-khod 123, Oman; 6 State Key Laboratory for Managing Biotic and Chemical Threats to the Quality and Safety of Agro-Products, Institute of Plant Virology, Ningbo University, Ningbo 315211, China

**Keywords:** disease, *Pantoea*, PGPR, rice, stress resilience

## Abstract

Bacteria from the genus *Pantoea* have been reported to be widely distributed in rice paddy environments with contradictory roles. Some strains promoted rice growth and protected rice from pathogen infection or abiotic stress, but other strain exhibited virulence to rice, even causing severe rice disease. In order to effectively utilize *Pantoea* in rice production, this paper analyzed the mechanisms underlying beneficial and harmful effects of *Pantoea* on rice growth. The beneficial effect of *Pantoea* on rice plants includes growth promotion, abiotic alleviation and disease inhibition. The growth promotion may be mainly attributed to nitrogen-fixation, phosphate solubilization, plant physiological change, the biosynthesis of siderophores, exopolysaccharides, 1-aminocyclopropane-1-carboxylic acid deaminase and phytohormones, including cytokinin, indole-3-acetic acid (IAA), auxins, abscisic acid and gibberellic acid, while the disease inhibition may be mainly due to the induced resistance, nutrient and spatial competition, as well as the production of a variety of antibiotics. The pathogenic mechanism of *Pantoea* can be mainly attributed to bacterial motility, production of phytohormones such as IAA, quorum sensing-related signal molecules and a series of cell wall-degrading enzymes, while the pathogenicity-related genes of *Pantoea* include genes encoding plasmids, such as the pPATH plasmid, the hypersensitive response and pathogenicity system, as well as various types of secretion systems, such as T3SS and T6SS. In addition, the existing scientific problems in this field were discussed and future research prospects were proposed.

## 1. Introduction

The *Pantoea* is a ubiquitous bacteria with a high degree of diversity and a variety of lifestyles, such as pathogen, epiphyte, endophyte and saprophytic, which has been often isolated from various geographical ecological niches and hosts, such as animals, humans and plants as well as some other environmental systems such as water and soil [1,2]. Indeed, the bacterial genus *Pantoea* is characterized by its functional versatility, environmental ubiquity and genetic similarity, and since the establishment of the genus with the type species *Pantoea agglomerans*, the ranks of the *Pantoea* genus have been strengthened by a large number of new species, which originated from a wide range of environments [3]. Nowadays, *Pantoea* is composed of 25 phenotypically similar species [4].

The *Pantoea* is defined as aerobic or facultative anaerobic, gram-negative, rod-shaped bacteria, which are typically motile by virtue of peritrichous flagella [2,5]. Bacteria belonging to the genus showed positive reactions for catalase, gelatin and starch hydrolysis tests [6,7,8], and are able to produce acid from the four carbon sources, including trehalose, palatinose, maltose and L-arabinose [9], but exhibited negative reactions for test of citrate utilization, nitrate, arginine dihydrolase, oxidation and sorbitol fermentation [5,10,11,12,13,14]. Colonies on nutrient agar medium were circular, smooth, mucoid convex with clear edges and yellow after 24 h of incubation at 28 °C [13]. The bacteria can grow in a wide range of temperature from 4 to 41 °C and pH from 2 to 8, while the optimum growth temperature and pH is 28–30 °C and pH 7, respectively [5].

Interestingly, *Pantoea* has been found to be widely widespread in rice environments. One hand, some studies showed that the application of *Pantoea* strains effectively improved rice growth and production due to their beneficial effects and potential to colonize in rice [15]. The growth promotion of rice by *Pantoea* may be mainly due to both direct nutritional acquisition and production of phytohormones, and indirect inhibition of rice plant pathogens, inducing broad-spectrum resistance or alleviating abiotic stress [16,17,18,19,20]. On the other hand, various reports have been published on the negative impact of *Pantoea* on rice production, which has been considered as an emerging rice pathogen, resulting in severe economic losses [21,22]. *Pantoea* obviously plays a controversial role in rice health, making it a current research hotspot. *Pantoea* is a kind of multi-faceted functional bacteria, but up to now, it is not still fully clear about the role of *Pantoea* in rice production.

In this review, we focus on the distribution, beneficial and harmful effect of *Pantoea* in rice production, focusing on its shift between the two types of roles. We also analyzed the mechanism underlying the beneficial role and the pathogenic risk of *Pantoea* spp. strains to rice growth, making it possible to differ the interaction of rice plants with the two types of *Pantoea* strains. In addition, the existing scientific problems in this field were discussed and future research prospects were proposed.

## 2. Distribution of *Pantoea* on Rice Plants

In recent years, the different species of *Pantoea* has been found to be closely related with rice-growth environments as an epiphyte, endophyte or even pathogen. In general, *Pantoea* spp. were found to be beneficial to the plant growth when they were reported as an epiphyte or endophyte of rice. This endophyte forms multicellular structures called symplasmata, which was able to alleviate the influences of other endobiotic organisms and change the inner environment during host plant growth, resulting into the improvement of the stability of endophytic *Pantoea* in the host plant [23]. Species of the genus *Pantoea* are often isolated from rice rhizosphere, rice phyllosphere, and various surface disinfected tissues (roots, stems, leaves and seeds), sampled from rice plants at different growth and development stages [24,25].

Although members of *Pantoea* are ubiquitous in various parts of the rice plants, the density is low at an early stage of rice growth and mainly comes from mature grains; however, at subsequent stages of rice, *Pantoea* is predominant and accounting for more than 80% of plant microbiota due to its highly aggressive capability [25,26]. Rice-associated *Pantoea* may be able to switch from the pathogenic lifestyle to a non-pathogenic or even beneficial lifestyle. However, it still remains unclear about the switch mechanisms. In order to differ between beneficial and pathogenic species of *Pantoea*, much research has been carried out to compare the interaction between rice plant and various strains of *Pantoea*. It has been proposed that the interaction of *Pantoea* and rice plants may be influenced by many factors, particularly the species of *Pantoea*, plant physiological status and climate environmental conditions [5,27,28].

## 3. Taxonomy and Phylogeny of *Pantoea* Species

*Pantoea* strains have been commonly isolated from different terrestrial and aquatic environments, as well as in association with plant, and animals [29,30]. The early taxonomy of members of *Pantoea* is quite complex, with some of the first members of the group being classified as *Enterobacter agglomerans* and *Bacillus agglomerans* [31]. Beji (1988) and Gavini et al. (1989) identified *Erwinia herbicola*, *E. milletiae* and *En. agglomerans* as also being synonymous, leading to the transfer of these three groups to the proposed name, *Pantoea agglomerans* [31,32], which served as the nomenclatural type for the establishment of the genus, *Pantoea*. Furthermore, *Pantoea* genus belonging to the family Enterobacteriaceae was first proposed in 1989 [33]. At that time, *Pantoea* only contained two species, *Pantoea dispersa* and *P. agglomerans*. Over the past few years, *Pantoea* species have expanded progressively [4,34,35]. The 25 currently recognized *Pantoea* species share many phenotypic characteristics with the high homology, which makes it difficult to identify the closely related species of *Pantoea* based on the API 20E, Biolog systems or the conventional PCR amplification of 16S rRNA fragments [10]. The reliable and accurate identification of *Pantoea* can be achieved by using several relatively expensive and time-consuming methods such as DNA–DNA hybridization and whole genome sequencing.

**Figure 1:**
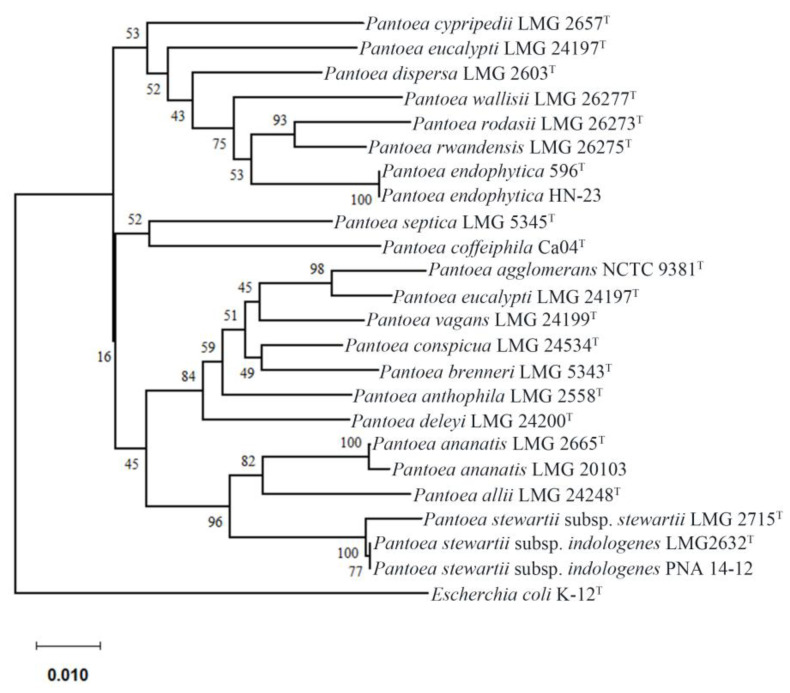
Neighbor-joining phylogeny of *Pantoea* type strains, based on a concatenated dataset composed of partial *atpD*, *gyrB*, *infB* and *rpoB* genes using maximum composite likelihood. Nodes show the result of 1000 bootstrap replicates.

**Figure 2:**
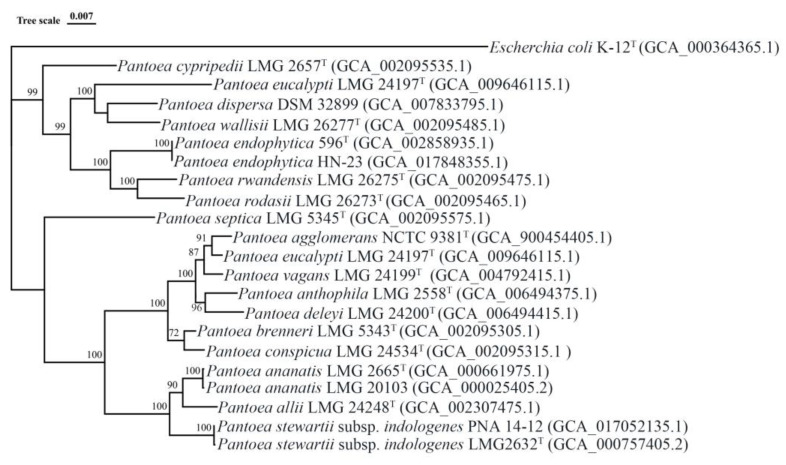
Phylogenetic tree of 18 species of *Pantoea*, based on 120 core genes using maximum composite likelihood. Nodes show the result of 1000 bootstrap replicates by IQ-TREE. Trees and dendrograms were visualized using ggtree and iTOL. Genomes from another strain were selected due to lacking the genomic information of the type strain of *P. intestinalis* and *P. dispersa,* while no genome information was available for *P. coffeiphila*.

Alternately, several techniques in particular multilocus sequence analysis (MLSA) have been used for the prompt and quick detection of *Pantoea* pathogens based on the sequence data from different housekeeping genes [36,37]. In this review, a rooted phylogenetic tree of *Pantoea* type strains that representative of each respective species was constructed using MEGA 7.0 based on the concatenated sequences of the four housekeeping genes *atpD*, *gyrB*, *infB* and *rpoB* (Figure 1). After that, we use IQ-TREE to build another rooted phylogenetic tree for the 120 core genes extracted from type strain genome to verify the MLSA result [38] (Figure 2). Interestingly, there was a high similarity between the phylogenetic tree of genome and that of MLSA. This revealed that the MLSA is a promising, prompt, reliable and quick method to differentiate the established *Pantoea* species.

## 4. Current Status of *Pantoea* Genome Analysis

Nowadays, more and more genomes of beneficial and virulent strains from rice have been successfully sequenced, which makes it possible to obtain genome-wide information for the pathogenicity and host specificity of *Pantoea*. The availability of these sequenced genomes from this highly versatile genus was able to help us better understand the metabolic characteristics of *Pantoea* and their colonization of host plants, as well as the underlying physiologic and genetic mechanisms that may contribute to the ability of certain isolates to thrive in different environments [39]. According to NCBI (https://www.ncbi.nlm.nih.gov/genome/?term=Pantoea), (accessed on 5 February 2022) the genomes of 636 *Pantoea* strains have been sequenced with sizes of 3.84–9.75 MB and G + C contents of 52.76–60.00%. The three most sequenced species are *Pantoea ananatis*, *P. agglomerans* and *Pantoea stewartii*, with 153, 123 and 27 strains, respectively. To date, the genome of 49 *Pantoea* isolates with pathogenic and beneficial and unknown roles from rice plants have been sequenced and mostly from *P. ananatis*, which was the most abundant and dominant species associated with rice plants [24].

The plant growth promotion ability can be supported by analysis of the *Pantoea*’s core genome, which contains a great number of genes that contribute to the beneficial functions of plants, such as nitrogen fixation, solubilization of inorganic phosphate, the biosynthesis of indole-3-acetic acid (IAA), siderophores and 1-aminocyclopropane-1-carboxylic acid (ACC) deaminase [1]. More recently, different types of siderophores produced by *Pantoea* spp. were justified by the fact that several *Pantoea* strains contain gene clusters involved in the biosynthesis of enterobactin-, desferrioxamine-, pyoverdine- and pyochelin-like siderophore based on a comparative genomic and phylogenetic analysis [40]. Banik et al. [41] identified the presence of *nifH* gene associated with nitrogen fixation in 2 *Pantoea* spp. strains from rice plants in India, which exhibited diazotrophic ability and increased the growth of rice seedlings.

**Figure 3:**
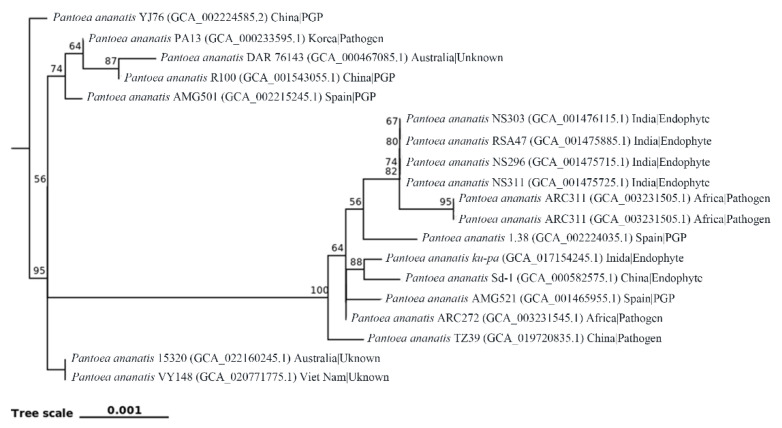
Phylogeny tree of *P. ananatis* strains associated with rice plants based on 120 core genes using maximum composite likelihood. Nodes show the result of 1000 bootstrap replicates by IQ-TREE. Trees and dendrograms were visualized using ggtree and iTOL. PGP: Plant growth promotion.

Generally, there was a high similarity in most of the biological properties between the virulent and environmental isolates of *Pantoea*, which could only be differentiated based on the pathogenicity. This result could be, at least partially, justified based on some common genomic features, such as both of them contain genes involved in plant growth promotion [42], virulence [43,44] quorum-sensing [45,46] and DNA repair and secretion systems [47]. In agreement with previous reports [36,39,48,49,50], the result of phylogenetic analysis in this study revealed that the 19 isolates of *P. ananatis* were unable to be differentiated based on the geological origin, beneficial or pathogenic role in rice growth (Figure 3).

The versatility and adaptability of *Pantoea* spp. are also reflected in several specific genetic features, such as the type VI secretion system (T6SS) and the Large Pantoea Plasmid family (LPP-1). A recent comparative genomic analysis showed that Pantoea isolates from diverse environments contain one T6SS variant involved in various roles such as antibiosis and plant pathogenicity [51]. Furthermore, LPP-1 ranged from 280 to 789 kb, was found to be common for all currently identified Pantoea species based on a comparative genomic study. The various roles of Pantoea in rice plants may be attributed to the Plasmid-encoded loci, which have been found to play a role in various bacterial functions such as virulence, antibiosis, host colonization, abiotic stress resistance, iron uptake and nitrogen assimilation as well as metabolism and transport of carbohydrates, amino acids and organic acids [44].

## 5. Beneficial Role on Rice Growth

Several studies have indicated that the beneficial effect of *Pantoea* on rice plants should be attributed to diverse mechanisms (Table 1), which include both direct growth promotion and indirect disease inhibition and abiotic alleviation [19,52,53].

### 5.1. Growth Promotion of Rice Plants

It has been reported from more than 10 years ago that *Pantoea* spp. significantly increased rice plants’ growth and yield [60]. For example, Zhang et al. [71] revealed a great potential for applying *Pantoea* spp. as an inoculant in rice production, which is the staple food of more than half the world’s population. Indeed, *P. agglomerans* could enhance the growth leaf, stem, and root hair as well as root elongation of rice plants [41,52], while *P. ananatis* significantly increased the growth and yield of rice plants by 60% [72]. Furthermore, Sun et al. [18] reported that *Pantoea alhagi* significantly increased fresh weight, root and shoot length of rice plants compared to the control.

Plant growth promotion of different *Pantoea* spp. may be mainly attributed to various mechanisms such as the biosynthesis of phytohormones, such as IAA, auxins, cytokinin, abscisic acid and gibberellic acid [52]. IAA has been reported to be able to play a dormant role in stimulating cell division, plant growth and differentiation [73]. Indeed, *P. ananatis, P. agglomerans, P. dispersa* and *Pantoea vegans* exhibited the ability to produce IAA [52,56,62,68], For example, Megías et al. [1] found that *P. ananatis* strain 1.19 from rice rhizosphere can efficiently produce IAA and increase plant production by 10% to 50% of rice and other cereals. Sergeeva et al. [74] isolated six *Pantoea* strains of IAA-producing bacteria with a plant growth-promoting potential.

The increased growth and yield of *Pantoea* species may also be due to the physiological change in rice plants. Indeed, the results of several studies have indicated that exposure of rice plants to *Pantoea* can result in a superior metabolism capacity inside plant cells. For example, *P. agglomerans* has been reported to significantly improve the photosynthetic characteristics and accumulation and transformation of assimilation products in rice plants compared to the control [52]. Furthermore, Sun et al. [17] found that there was a 26.3% increase in chlorophyll content when rice roots were colonized by *P. alhagi*, which also caused an up-regulation of proline synthase expression and down-regulation of proline dehydrogenase expression, as well as the increase in antioxidant enzyme activities compared to the negative control plants.

Meanwhile, several studies have also stated that *Pantoea* can synthesize siderophores, improving iron utilization rate and promoting plant growth by chelating trivalent iron in the environment [70]. For example, *P. ananatis* AMG501 and AMG521 all have the capacity to synthesize siderophores and increases plant growth and crop yield significantly [1,57]. Loaces et al. [25] identified six strains of *Pantoea* that can synthesize siderophores and promote rice growth. *Pantoea* spp. are quite competitive among the siderophores-producing bacteria, among which *P. ananatis* were permanently associated with rice tissues. In addition, many researches proved *Pantoea* could produce ACC deaminase [10,19], which could protect plants by reducing the high concentration of ethylene [75]. Lu et al. [20] reported that *P. ananatis* D1 has strong ability in ACC deaminase production and enhanced the growth of rice plants under normal and saline conditions. Yang et al. [19] isolated the 9 *Pantoea* strains and found that all strains have a good ability of ACC deaminase synthesis, suggesting that ACC deaminase synthesis may be a common ability of *Pantoea*.

Furthermore, nutrient-related promoting traits, particularly the ability to solubilize phosphate or fix nitrogen were described in most *Pantoea* strains [42]. For example, Verma et al. [58] isolated endogenous nitrogen-fixing *P. agglomerans* from rice and found it has a high growth-promoting potential for rice growth. Feng et al. [52] observed that rice endophyte *P. agglomerans* YS19 is a typical diazotrophic endophyte, which not only exhibited the activity to fix nitrogen in N-free medium, but also could improve rice growth by increasing root elongation and the biomass of leaf, stem and root hair of rice plants under the condition of nitrogen deficiency. Ghosh et al. [62] reported that *P. dispersa* AS18 isolated from agricultural land displayed nitrogen fixation and phosphate solubilization, which could be used to improve rice production under abiotic stress. Li, et al. [76] revealed that *P. agglomerans* ZB could significantly increase the content of available soil phosphorus and potentially improve plant growth. Bakhshandeh et al. [56] experimentally proved that *P. ananatis* M36, isolated from rice paddy soil, has a good ability of phosphate solubilization activity and could be used as inoculants to promote the growth of rice plants. In addition, some *Pantoea* species such as *P. agglomerans* produced phytase (phytate-degrading enzyme), which are key for making this rich phosphorus source in the rhizosphere and other soil layers is available to plants [15].

### 5.2. Alleviation of Abiotic Stress

Some *Pantoea* strains could improve rice seedlings growth by alleviating some abiotic stress, such as heavy mental, salt and drought. For example, *Pantoea* spp. EA106 promotes rice development, and reduces the accumulation of toxic arsenic (As) in plant tissue [70]. Similarly, *P. dispersa* strain AS18 could reduce as uptake with a simultaneous improvement in seedling growth, chlorophyll contents and the activities of antioxidant-related enzymes [62]. In another study, Tian et al. [77] reported that the endophytic bacteria *P. agglomerans* Tm02 improved the plant biomass plants in Cd-contaminated soil, and reduced the Cd concentration in rice grains. Likewise, Zhou et al. [78] revealed that the inoculation of *P. agglomerans* R3-3 significantly ameliorate Cd contamination in paddy fields and can be a better alternative for the safe rice production. Moreover, the endophytic bacterium *P. alhagi* NX-11 has been found to be able to alleviate the damage of salt and drought stress to rice seedlings by increasing the K^+^/Na^+^ ratio, the activities of antioxidant-related enzymes including catalase, peroxidase and superoxide dismutase, the content of total proline, chlorophyll and soluble sugar, and decreasing the malondialdehyde content [17,18,79].

Systemic abiotic tolerance was also often induced when the *Pantoea* species colonized roots of rice plants. For example, *P. agglomerans* stimulated rice plants growth under the conditions of poor soil [80]. Bhise and Dandge [81] found that *P. agglomerans* reduced sodium uptake and the level of proline and malondialdehyde, but increased the length, biomass and photosynthetic pigment, as well as calcium and potassium uptake under salt stress conditions, indicating that this bacterium has a significant growth improvement potential in rice plants. Meanwhile, NaCl and Na_2_CO_3_ have been reported to induce oxidative stress in rice, which can be ameliorated by *P. ananatis* [20]. Recently, Ghosh et al. [62] showed that *P. dispersa* exhibits the ability to reduce the uptake of arsenic and the levels of ethylene in plants, but enhance the growth of rice seedling. For some isolates of *Pantoea* spp., the successful colonization and survival may be, at least partially, attributed to the production of IAA and the carotenoids, which has been found to be involved in the nutrient leakage of plant leaves and protection of the cells from UV exposure and UV-activated reactive oxygen species [39,82,83,84].

### 5.3. Protection of Rice Plants from Pathogen Infection

A large number of studies indicated that *Pantoea* spp. has a great potential to be used as effective biocontrol agents for inhibiting rice important pathogens and controlling various rice bacterial and fungal diseases such as rice bacterial leaf blight, rice blast through antibiosis production, niche competition or induced resistance [67,69,85]. For instance, Yang et al. [19] found that *Pantoea* strains isolated from diseased rice leaves exhibited the in vitro and in vivo inhibition in the growth of *Xanthomonas oryzae* pv. *oryzae* (Xoo). Furthermore, *P. ananatis* and *P. agglomerans* had been shown to have in vitro and in vivo inhibitory activity against *Magnaporthe grisea* (anamorph: *Pyricularia grisea*), *Magnaporthe oryzae* [22,64,66].

The *Pantoea* strains have been regarded as an antagonist of many plant bacterials and fungal pathogens by producing a variety of extracellular hydrolytic enzymes (cellulase, chitinase and glucanase, protease) or antibiotics, such as pantocins (A and B), herbicolins (A, B and I), agglomerins (A, B, C and D), andrimid, microcins and phenazines, D-alanylgriseoluteic acid (AGA), 2-amino-3-(oxirane-2,3-dicarboxamido)-propanoyl-valine, *Pantoea* Natural Product (PNP-1, 2, 3, 4), which effectively protect rice from various pathogen infections [10] (Table 2). For example, *P. ananatis* exhibited more than 50% biocontrol efficacy against rice blast pathogen *M. grisea* under both greenhouse and field conditions by secreting extracellular hydrolytic enzymes [67]. Similarly, Azman et al. [68] isolated two *Pantoea* strains, which could produce hydrolytic enzymes and show antagonistic activity against rice pathogen Xoo.

In addition to direct inhibition, *Pantoea* was also able to inhibit the cell growth of pathogenic bacteria and other microbes through nutrient and spatial competition [86]. Pasichnyk et al. [87] reported that *P. agglomerans* can effectively control plant disease through quicker propagation than the pathogen. As we know, *Pantoea* is an endophytic colonizer of its rice host, which has been found to be more aggressive than other rice-associated bacteria. For example, the colonization of *Ochrobactrum* sp. on rice plants was markedly inhibited when co-inoculated with the equal number (10^5^ CFU/mL) of GFP-tagged *Pantoea* sp. and *Ochrobactrum* sp.; however, the colonization of *Pantoea* sp. on rice plants was unaffected by *Ochrobactrum* sp. [88].

As well as antibiosis and competition, the induction of plant systemic resistance may also be involved in protecting *Pantoea* for rice plants from disease infection. For instance, Ortmann et al. [16] proved that the extracellular polysaccharide of *P. agglomerans* could enhance the defense response of rice plants to infection of the pathogens. Similarly, Spence et al. [69] showed that the defense response elicited by *P. agglomerans* isolated from rice rhizosphere is mediated through the signaling pathways of both jasmonic acid and ethylene of rice plants. Furthermore, Ortmann et al. [16] revealed that exopolysaccharides (EPSs) of *Pantoea* can induce the resistance of rice plants to disease by potentiating the defense response elicited by the infection of the pathogen with the generation of H_2_O_2_ defined as an ‘oxidative burst’.

**Table 2:** Type of antibiotic produced by *Pantoea* species.

Antibiotic	Target Pathogen	Species/Strain	Origin	Reference
AGA	Gram-positive pathogens	*P. agglomerans* Eh1087	Apple; New Zealand	[89,90]
Andrimid	MRSA; VRE; *Kp* and human tumor cell lines	*P. agglomerans*	-	[91]
APV	*Ea*, Psg, *At*, *Ca*	*P. agglomerans* Pa48b/90	Soybean, Germany	[92]
Agglomerins A, B, C, D	*Cd*; *Cp*; *Pa*; *Sp* and Spyo	*P. agglomerans*	-	[93]
Herbicolin A and B	Sterol-containing fungi	*P. agglomerans* A111	Gramineae; Germany	[94,95]
Herbicolin I	*Ea*	*P. vagans* C9-1	Apple; USA	[96]
Microcin	*Ea*	*P. agglomerans* Eh252	Apple; USA	[97]
PNP-1	*Ea*	*P. ananatis* BRT175	Strawberries-	[98,99]
PNP-2	*Ea*; *Ec*; *Enterobact*; *Klebsiella*, *Kosakonia; Pseudocitrobacter; Salmonella; Staphylococcus; Streptococcus* and most *Pantoea* strains	*P. agglomerans* Tx10	Clinical, USA	[100,101]
PNP-3	*Ab*, *Pa*	*P. agglomerans* 3581	Oat; ICMP	[102,103]
		*P. agglomerans* SN01080	Slug, Canada	[50,102]
PNP-4	*Enterobacter* and *Kosakonia*	*P. agglomerans* B025670	Human; Canada	[104]
Pantocin A	*Enterobacteriaceae* strains	*P. agglomerans* Eh318	Apple; USA	[105]
		*P. agglomerans* P10c	Apple; New Zealand	[97]
		*P. agglomerans* Tx10	Clinical, USA	[100,101]
		*P. vagans* C9-1	Apple; USA	[106]
		*P. brenneri* LMG 5343	Human; USA	[49]
Pantocin B	*Enterobacteriaceae* strains	*P. agglomerans* Eh318	Apple; USA	[105]
Phenazine	*Cm*, *Ba*, *Cb*, *Dz*, *Pc*, *Pp*, *Se*, *Ec*, *Kp*, *Ye*	*P. agglomerans* R190	Apple; Korea	[107]

Ab: *Acinetobacter baumannii*; AGA: D-alanylgriseoluteic acid; APV: 2-amino-3-(oxirane-2,3-dicarboxamido)-propanoyl-valine; At: *Agrobacterium tumefaciens*; Ba: *Burkholderia andropogonis*; Ca: *Candida albicans*; Cb: *Chryseobacterium balustinum*, Cd: *Clostridium difficile*; Cm: *Clavibacter michiganensis*, Cp: *Clostridium perfringens*; Dz: *Dickeya zeae*; Ea: *Erwinia amylovora*; Kp: *Klebsiella pneumoniae*; MRSA: methicyllin-resistant *Staphylococcus aureus*; Pa: *Propionibacterium acnes*; PNP-1: *Pantoea* Natural Product 1; PNP-2: *Pantoea* Natural Product 2; PNP-3: *Pantoea* Natural Product 3; PNP-4: *Pantoea* Natural Product 4; Psg: *Pseudomonas syringae* pv. (pathovar) *glycinea*; Sp: *Streptococcus pneumoniae*; Spyo: *Streptococcus pyogenes*; VRE: vancomycin-resistant *Enterococcus*; Ec: *Escherichia coli*; Pa: *Pseudomonas aeruginosa*; Pc: *Pectobacterium carotovorum*, and Pp: *Pseudomonas putida*, Se: *Salmonella enterica*; and Ye: *Yersinia enterocolitica*.

## 6. Risk on Rice Production

Rice is a main source of food for a large part of the world’s human population. Unfortunately, some species of *Pantoea* have been reported to be one of the causal agents of rice diseases, which makes it have been regarded as a future threat to the production of rice. Thus, it is very necessary to know the risk of *Pantoea* spp. in rice production.

### 6.1. Virulence of Pantoea Species to Rice

Although bacteria from the genus of *Pantoea* is usually present in rice plants as an epiphytic or endophytic, two *Pantoea* species (*P. ananatis* and *P. agglomerans*) have been widely reported as a type of opportunistic pathogens to rice plants in Australia, Italy, China, Korea, Russia and Brazil in last two decades. In general, the two species exhibited a weak virulence to rice tissue with the symptoms such as grain discoloration, the reduced germination of seeds, stem necrosis, palea browning and sheath rot (Table 3). For example, Egorova et al. [7] observed the grain discoloration caused by *P. ananatis* initially caused light, rusty, water-soaked lesions, which later turned brown, to appear on the plant lemma or palea. In another study, Carrer Filho et al. [108] reported that *P. agglomerans* is associated with germplasm of rice seeds with lower germination.

To our surprise, *Pantoea* spp. has recently been identified as the causal agent of a new rice bacterial disease with up to 70% incidence in susceptible rice varieties, causing BLB-like symptoms [21]. In addition, some certain *Pantoea* strains are also major disease agents of edible fungi (mushroom production) [109,110]. In some rural areas, the wastes and residues of mushroom media are used as organic fertilizers [111,112], which might be new sources of rice pathogens. The first observations were water-soaked lesions and then along the leaf blades showing a light brown and blighted appearance. The incidence of this disease is highly associated with bacterial strains, rice cultivar and the environment of rice fields. The pathogenic *Pantoea* can enter rice host through flowers, wounds caused by feeding insects, mechanical damage and plant contact during strong winds [22], while in severe cases, this new bacterial disease resulted in the incidence of 20 to 100% yield loss in rice [9], which makes the genus *Pantoea* regarded as one of the next major phytopathogenic rice species. Nowadays, this new rice disease caused by *Pantoea* has been reported in many counties including China, Malaysia, Germany, Turkey, Togo, Korea, India, Thailand, Brazil, Venezuela, and Tamil Nadu. The pathogen of this disease has been attributed to *P. ananatis*, *P. stewartii*, *P. agglomerans* and *P. dispersa*, while *P. ananatis* seems to be the main pathogenic species, which makes the *Pantoea* genus regarded as to be a devastating threat to rice production worldwide, thus resulting in severe losses in the yield and quality of rice. In addition, the disease was caused by two species complexes in some counties such as Germany, Togo, Malaysia and Thailand, indicating the complexity of this causal agent [4].

**Table 3:** Summary on the virulence of *Pantoea* to rice from reported countries.

Symptoms	*Pantoea* Species	Strains	Isolated from	Reference
BLB-like disease	*P. ananatis*	ITCC B00-50/-55	India	[12]
		17671	Benin	[113]
		ARC-60/-651	Togo	[114]
		PaTo34a1	Turkey	[14]
		PA-1/-3/-5 to 12	Malaysia	[115]
		PA	Malaysia	[116]
		-	Thailand	[117]
		SC7	China	[118]
		FY43, JH-31/-99, TZ-20/-39/-68	China	[72]
	*P. stewartii*	626	Benin	[113]
		ARC-229/-646	Togo	[114]
		TVL-ASD/-TN 1	India	[119]
		PRE17_104	Thailand	[117]
		MF1 to 5, MF7 to 9	Malaysia	[8]
	*P. dispersa*	PC	Malaysia	[116]
	*P. agglomerans*	EMLORY-1 to 4	Korea	[6]
		A-1/-2	Venezuela	[9]
		PagK35b	Turkey	[13]
Stem necrosis	*P. ananatis*	ICMP 1580	Australia	[120]
	*P. agglomerans*	ICMP 272	Australia	[120]
Palea browning	*P. agglomerans*		Japan	[121]
	*P. agglomerans*	-	Korea	[122]
	*P. agglomerans*	-	China	[123]
	*P. ananatis*	-	Japan	[124]
	*P. ananatis*	-	Japan	[125]
	*P. ananatis*	-	Italy	[126]
sheath rot	*P. ananatis*	PA13	Korea	[127]
Grain discoloration	*P. ananatis*	N-1-1, O-2-2 and C-2-3	China	[128]
		AIMST 1.Po.15	Russia	[7]
	*P. agglomerans*	FDQ1, FDSN4, XD2 and XSH4	China	[129]
	*P. ananatis*	MAFF 301720	Japan	[121]
		-	Russia	[7]
		-	China	[128]
Seed dormancy	*P. agglomerans*	Bac-1887/-2821/-2926/-2935	Brazil	[108]
No germination of seeds	*P. agglomerans*	Bac-1887/-2821/-2926/-2935	Brazil	[108]

### 6.2. Pathogenic Mechanism

Several virulence determinants have been reported to be highly associated with the plant pathogenicity of *Pantoea*. For example, the pathogenesis of *P. ananatis* may be related with the produced IAA [24,130,131,132]. Meanwhile, motility plays a crucial role in the location and attachment of *P. ananatis* to plant leaf surfaces efficiently [133]. Furthermore, Ma et al. [134] reported that *P. ananatis* can produce several cell-wall degrading enzymes, which may participate in pathogenicity by effectively degrading rice straw, cellulose, hemicellulose and lignin, thereby helping bacteria invade plant cells and infect host tissues. Moreover, the pathogenicity of *Pantoea* to rice plants may be due to the production of signal molecules associated with quorum sensing, which has been shown to play a role in bacterial pathogenicity, biofilm formation and biosynthesis of EPSs and the biosynthesis of hydrolytic enzymes [2].

Several other mechanisms have been recently proposed for the virulence of *Pantoea* to rice plants. The pathogenicity-related genes of *Pantoea* are often located in plasmids, such as a pathogenicity island in the pPATH plasmid of *P. agglomerans* [135]. Furthermore, the pathogenicity of *Pantoea* may be also attributed to the phytohormones, hypersensitive response and pathogenicity (*hrp*) system, as well as various secretion systems, such as T6SS in *P. ananatis* [10] and type Ⅲ secretion system (T3SS) in *P. agglomerans* and *P. stewartii* [39]. These secretion systems have been found to be the important determinants of virulence by delivering effector proteins directly to the host cell or host environment, thereby achieving pathogens’ successful colonization and growth [136]. The specific interaction between the bacterium and host can be facilitated by activation of the T3SS, which causes the injection of T3SS effectors into the host cell [137]. The T3SS has been reported in most of the *Pantoea* species; in contrast, comparative genomics analysis revealed the absence of the Type II, III and IV secretion systems, but the presence of T6SS in *P. ananatis*, revealing the complexity of pathogenesis in *Pantoea* species [51]. Indeed, a lot of pathogenicity and virulence genes of *P. agglomerans* have been identified or predicted (Table 4), which help us better understand the pathogenic mechanism.

## 7. Mechanism for Various Roles in Rice Plants

Some *Pantoea* strains can promote rice growth, but others can cause disease on rice, indicating the diversity and complexity of *Pantoea*. The shift of *P. agglomerans* from saprophytic lifestyle to pathogenic lifestyle has been partially attributed to the gain of a plasmid-borne pathogenicity island (PAI) that contains the cluster of *hrp*/*hrc* gene [139,146]. More recently, Hofmeister et al. [147] showed that plant pathogenic strains of *P. ananatis* contained the genes involved in the production of an N-formylated sugar on the O-antigen, which, apparently, the non-pathogenic strains of *P. ananatis* did not have, based on the results of bioinformatics analysis; however, it is still unclear as to the role of this N-formylated sugar in bacterial virulence. In contrast, environmental, beneficial and pathogenic isolates of *P. ananatis* were unable to be clustered into each respective species group, but were rather intermingled, with environmental isolates possibly having a potential to colonize plant hosts. In addition, the substantive recombination between *P. agglomerans* isolates has been discovered based on the analysis of split decomposition [48], suggesting a transfer of genetic determinants has occurred between individual isolates with different biological functions.

In general, there is little information about the ecological adaption of *Pantoea* to various niches and living environments. Indeed, our previous study indicate that various role of bacteria from the same species may be mainly due to its niche adaptation [148]. Studies have shown that the great variability of *Pantoea* isolates is mainly due to the plasticity of its genome, particularly the LPP-1, which derived from a common ancestor and has undergone extensive diversification among the *Pantoea* [44]. The LPP-1 has been regarded as an important driver of the biological, ecological and lifestyle diversification, which was observed among several *Pantoea* species [44]. The genes carried by this plasmid can confer various phenotypes on bacteria, including hormone production, iron and nitrogen assimilation and pathogenicity [44]. Thus, the growth promotion and disease-causing *Pantoea* may be due to the presence of LPP-1 plasmids, which represent the main evolutionary driving force of the ecologically and biologically adaptation. Therefore, further study needs be carried out to understand the interaction between *Pantoea* and rice plants, which will help us elucidate under which circumstances *Pantoea* plays a role in promoting growth on rice, and in which environment *Pantoea* infects rice growth and cause disease.

## 8. Safety of *Pantoea*

More and more attention has been paid on the safety of *Pantoea* strains. Indeed, based on the Committee on Biological Agents (2015), only four species *P. agglomerans*, *P. brenneri, Pantoea eucrina* and *Pantoea septica* are not listed as risk group 1 from the 27 species listed on the TRBA-466, while 11 species have been regarded as plant pathogens. On the other hand, *P. agglomerans* has been reported to be used as adjuncts to agricultural practice at commercial level [149]. Furthermore, two commercial biocontrol products, Blight-Ban C9-1 and Bloomtime Biological, have been developed by *P. vagans* strain C9-1 and *P. agglomerans* strain E325 with the ability to produce antimicrobials, respectively, while the commercial biocontrol products exhibit great potential in the control of fire blight infection of apple and pear trees [150,151,152,153].

## 9. Detection and Differentiation of *Pantoea* Spp.

There was an increasing application of molecular approaches in identifying bacterial pathogens since it is faster, more specific, and more accurate. In general, rice isolates of *Pantoea* were often identified based on conventional PCR amplification using universal 16S rDNA fragment. However, many pairs of specific primers for *Pantoea* species, particularly the pathogenic species, have been designed based on the highly conserved housekeeping genes (e.g., *gyrB*, *rpoB atpD* and *infb*) [36]. Indeed, Carrer Filho et al. [108] successfully distinguished the rice *Pantoea* pathogens using three specific primers pairs. Furthermore, compared with conventional PCR, a multiplex PCR (mPCR) has been recently developed by Kini et al. [4], which could be used as a diagnostic tool for quick and accurate detection of three major rice pathogenic *Pantoea* spp. including *P. ananatis*, *P. stewartia* and *P. agglomerans* simultaneously.

On the other hand, we summarized the physiological and biochemical properties of three major pathogenic *Pantoea* spp. in rice plants [5,8,72,115,119], which can be used to detect and differentiate *Pantoea* species associated with rice diseases. Results showed positive reactions towards hydrolysis of starch and catalase, and negative reactions for oxidase reaction and hydrogen sulphide production. However, they differed in indole production, gelatin liquefaction, nitrate reduction, citrate utilization and phenylalanine deaminase (Table 5). Detection of phenotypic and molecular-based approaches provide us a better insight for understanding the diversity of *Pantoea* spp. and helps us develop effective strategies to control this emergent bacterial disease of rice.

### Differentiation of Beneficial and Pathogenic Strains

Nowadays, many studies have characterized the *Pantoea* strains with pathogenic and beneficial roles. However, the similarity of *Pantoea* genomes is very high, while they may only be able to be differed based on the gentle differences in plant pathogenicity. De Maayer et al. [154] observed that there are 89.3 to 95.7% of the proteins common among the 8 strains of *P. ananatis*. Furthermore, comparative genomics analysis indicated that three *P. agglomerans* strains are highly conserved and there are no significant differences between them in bacteriological characteristics, only small differences in genes encoding T6SS, phage/transposase/integrases, and eukaryotic-like protein domains [5,155]. Although protein studies confirmed the presence of the hemolysin co-regulated effector proteins (Hcp) protein that is associated with bacterial motility, biofilm formation and protease production in the growth-promoting strain S6, and the absence in the plant-pathogenic strain S7, its role in determining the beneficial effect of strain S6 and pathogenic effect of strain S7 remains to be understood [155].

In contrast, results from this review indicated that beneficial and pathogenic *Pantoea* strains that isolated from rice plants do not form distinct populations based on the analysis of all the currently obtained *P. ananatis* genomes downloaded from the NCBI database (Figure 3). This is consistent with a variety of previous studies [156], which revealed the indistinguishable virulence potential in both clinical and plant *P. agglomerans* isolates. Currently, the two types of strains were only distinguished based on the pathogenicity of rice seedling. Therefore, further research needs be carried out to find the difference between pathogenic and non-pathogenic strains, which not only will provide a clue for us to understand the pathogenic mechanism, but also give a guide for the utilization of beneficial strains in rice production.

## 10. Conclusions

In conclusion, it has been well documented that there was a high abundance of the *Pantoea* spp. in rice environments, while different strains within the *Pantoea* genus may play opposite roles by either promoting rice growth or causing disease, indicating that the role of *Pantoea* in rice plants is very complex. Currently, it is still unclear why strains with the same species have various roles in rice plants; however, the universality and versatility of *Pantoea* strains make it a good model organism to explore specific niche adaptations and develop commercial agricultural products. The interaction of rice plants with *Pantoea*, may be determined by many factors, such as specific strains of *Pantoea* with either beneficial or pathogenic roles, the environmental fitness and plant physiological status. Nowadays, more and more attention has been paid to understanding the mechanism of *Pantoea* for its various roles on rice plants, which is undoubtedly a research area worthy of further research.

## Figures and Tables

**Table 1:** Beneficial effect of Pantoea species in rice growth promotion and disease suppression.

Beneficial Effects	*Pantoea* Strains	Main Mechanisms	Applications	Reference
Growth promotion	*P. ananatis* 1.38	P-solubilization; siderophores production; IAA production	Rhizospheric application	[54]
	*P. ananatis* 9C	N-fixation	Rhizospheric application	[55]
	*P. ananatis* M36	P-solubilization; IAA production	-	[56]
	*P. ananatis* AMG521	P-solubilization; IAA and siderophores production	Rhizospheric application	[53]
	*P. ananatis* AMG 501	IAA and siderophores Production	Rhizospheric and foliar application	[57]
	*P. ananatis* D1	P-solubilization; IAA, siderophores and ACC deaminase production	Rhizospheric application	[20]
	*P. agglomerans*	N-fixation; IAA and salt tolerance siderophores production	Rhizospheric application	[58]
	*P. agglomerans* HK 14-1	P-solubilization	-	[59]
	*P. agglomerans* YS19	N-fixation; IAA production	Foliar spray	[52]
	*P. agglomerans* PaJ and BS2a	P-solubilization	Seeds soaking	[60]
	*P. alhagi* NX-11	Salt/drought resistance	Rhizospheric application/Foliar spray	[17,18]
	*P. rodasii* S32	P-solubilization	Rhizospheric application	[61]
	*P. dispersa* AS18	N-fixation; P-solubilization; IAA; ACC deaminase production and AS resistance	Seeds soaking	[62]
	*Pantoea* sp. SB19, WR23	N-fixation; IAA production	Rhizospheric application	[41]
	*Pantoea* sp. 1.19	IAA, siderophores and ACC deaminase production	Rhizospheric and foliar application	[1]
	*Pantoea* sp.	IAA	-	[63]
Disease suppression	*P. ananatis* NR-1	Endochitinolytic enzyme cloned from *Serratia marcescens* B2	Foliar spray	[64]
	*P. ananatis* R100	Oxazolomycin and chalcomycin	-	[65]
	*P. agglomerans*	antifungal compounds	-	[66]
	*Pantoea* sp. HS-8	extracellular hydrolytic enzymes and siderophores production	Foliar application	[67]
Growth promotion and disease suppression	*P. vegans* LBB2 and *Pantoea* sp. LBC1	N-fixation; P-solubilization, IAA and antibiotics production	-	[68]
	*Pantoea* spp. M18, M11, E3, L42	Siderophores, N-fixation and IAA production	-	[25]
	*Pantoea* sp. EA106	Siderophores, induced resistance, As-resistance,	Rhizospheric application	[69,70]

N: nitrogen; P: Phosphate.

**Table 4:** The pathogenicity and virulence genes annotated in the genomes of *P. agglomerans*.

Pathways	Virulence Genes	Annotation	References
Virulence factor	*hvaA*	Hypothetical	[138]
Secretion system	*TssA, TssB, TssC, TssD (Hcp), TssE, TssF, TssG, TssH (ClpV), TssI (vgrG), TssJ, TssK, TssL (DotU)*, *TssM (IcmF), tagF, tagH, PAAR*	Type VI secretion protein	[138]
	*hrpJ, hrpN, hrpY*	HR and pathogenicity genes	[139,140]
	*HsvG, hsvB, HopAF1, HopD1, HopR1, HopX2, HopAY1, 1611, PthG, PseB, 1595, HrpN, HrpK, HopV1, HopAK1, 2716, 2223, 585, 1337, 2073, 2097, 2728, 3721, 2591*	Type Ⅲ secretion protein	[141]
Adhesion	*fha*	Filamentous hemagglutinin	[138]
	*ompA*	Outer membrane protein A	
Motility	*fliC, fliD, fliE, fliF, fliG, fliH, fliI, fliJ, fliK, fliL, fliM, fliN, fliO, fliQ, fliS, fliT, fliZ, flhA, flhC, flhD, flhE, flgA, flgB, flgC, flgD, flgE, flgF, flgG, flgH, flgI, flgJ, flgK, flgL, flgN, motB*	Flagella	[140]
	*CheV, CheY, CheW, CheA*	Chemotaxis protein	
Iron uptake system	*fur*	Ferric iron uptake^break/^transcriptional regulator	[139]
	*EfeO*	Iron uptake system protein	
	*SitC*	Iron/manganese ABC transporter permease subunit	
	*fepB, fepG, entS*	Enterobactin transporter	
	*fepA*	TonB-dependent siderophore receptor	
	*fepD*	Ferric siderophore ABC transporter permease	
	*fhuF*	Siderophore-iron reductase	
Toxin	*Hha, ShlB, FhaC, HecB, XhlA*	Hemolysin	[139]
	*hlyIII*	Hemolysin III	
	*HicA, HicB, YefM, EF hand domain protein*	Toxin-antitoxin(s)	[142]
Host specificity	*pyhG*	Avr-like protein	[139]
Changing the defense signal of host	*avrxacE2, avrxacE1,*	*avr* genes	[143]
Induce necrosis	HiVir	High virulence protein	[144,145]
Tolerance	*alt*	Allicin tolerance	[144,145]
	*acrAB*	periplasmic membrane-fusion protein and inner membrane protein	[143]
Cell wall-degrading enzyme	*kdgM*	Oligogalacturonate specific porin	[142]

**Table 5:** Biochemical tests of Pantoea species associated with rice plants.

Test	*P. ananatis*	*P. agglomerans*	*P. stewartia* Subsp. *indologenes*
Gram reaction	*-*	*-*	*-*
Motility	*+*	*+*	*+*
Indole production	+	−	+
Oxidase reaction	−	−	−
Hydrolysis of starch	+	+	+
Gelatin liquefaction	+	−	+
Nitrate reduction	−	+	−
Citrate utilization	−	−	+
Catalase	+	+	+
Phenylalanine deaminase	−	+	−
Hydrogen sulphide production	-	-	-

+: positive reaction; −: negative reaction.

## Data Availability

Not applicable.
